# Case Report: A case of surgical and enzyme replacement therapy for type I Gaucher disease complicating femoral shaft pathological fracture

**DOI:** 10.3389/fsurg.2025.1616941

**Published:** 2025-08-22

**Authors:** Yang Gao, Jia Liu, Zhijie Zhang, Xinhao Sheng, Yuerong Wang, Xin Zhao, Huanzhi Ma

**Affiliations:** ^1^ Department of Orthopaedics, Shandong Provincial Hospital Affiliated to Shandong First Medical University, Jinan, China; ^2^ School of Clinical Medicine and Basic Medical Science, Shandong First Medical University & Shandong Academy of Medical Sciences, Jinan, China; ^3^ School of Ophthalmology, Shandong First Medical University & Shandong Academy of Medical Sciences, Jinan, China

**Keywords:** Gaucher disease, open reduction internal fixation, enzyme replacement therapy, L444P, A170H

## Abstract

Gaucher disease (GD) is an inherited lysosomal storage disorder caused by glucocerebrosidase (GCase) deficiency. A 35-year-old male patient was admitted to our hospital due to left thigh pain and restricted mobility for 10 h. Following comprehensive evaluations, the patient was diagnosed with GD complicated by a pathological fracture of the left femur. He has a known L444P mutation, a suspected pathogenic A170H mutation, and an A271 V mutation of uncertain significance not in GD databases. However, a potential association with the disorder cannot be excluded. We speculate that the patient's marked thrombocytosis may be related to the rare A170H and A271 V mutations. After an assessment, a decision was made to perform curettage of the left femoral lesion and open reduction internal fixation (ORIF) for the fracture. Postoperative management included ongoing enzyme replacement therapy. To date, case reports of GD patients undergoing ORIF for fractures are relatively rare, and the patient in this case harbored rare A170H and A271 V mutations. We report this case with the aim of sharing experience related to internal fixation for fractures in patients with GD, summarizing the specific phenotypes presented by specific gene mutation types, and providing a basis for the subsequent discovery of new gene mutations in GD.

## 1 Introduction

Gaucher disease (GD), one of the most prevalent lysosomal storage disorders, has heterogeneous global incidence rates attributable to geographical and ethnic disparities. Although its prevalence varies significantly across populations, GD profoundly impairs the quality of life of affected patients and their families while imposing a substantial burden on public health systems worldwide. In addition, referred to as glucocerebrosidosis (GCase), GD is an autosomal recessive genetic disorder arising from mutations in the *GBA* gene, resulting in reduced GCase activity (1). This molecular defect leads to the pathological accumulation of glucocerebroside in the mononuclear-macrophage system, thereby inducing multisystemic involvement (2). GD has a relatively high prevalence in Jewish populations and a relatively low incidence among individuals of East Asian descent. Following its first description in 1882 by the French dermatologist Philippe Gaucher (3), extensive research has gradually improved our understanding of its pathogenesis, clinical phenotypes, and therapeutic strategies. In the early stages, limited by technological constraints, the disease can only be preliminarily classified on the basis of clinical manifestations. Pathogenic genes and associated metabolic pathways have been progressively elucidated with the advent of molecular biology techniques.

GD is categorized into three distinct clinical phenotypes—type I (nonneuronopathic), type II (acute neuronopathic), and type III (subacute neuronopathic)—on the basis of the age of onset and the degree of organ system involvement (4). The early diagnosis of GD remains challenging due to the heterogeneity of initial clinical manifestations and the fragmentation of healthcare settings where patients first seek treatment. As of 2023, the diagnostic landscape for GD in China remains suboptimal, with a reported misdiagnosis rate of 37% and 31% of patients experience delays in obtaining a diagnosis within the same year of symptom onset. Enhancing diagnostic accuracy for GD is therefore a critical unmet need. Here, we present a case of GD type I complicated by a pathological fracture of the left femur. Postoperative management included imiglucerase enzyme replacement therapy (ERT), which yielded favorable clinical outcomes. Genetic sequencing further revealed *GBA*-associated mutations consistent with GD pathogenesis. This case report aims to augment the understanding of the complex clinical presentations of GD, deepen insights into its molecular mechanisms, and provide valuable clinical insights for similar cases.

## 2 Patient information

A 35-year-old male patient who presented to the emergency department with a 10-hour history of left thigh pain and limited mobility denied significant trauma. Initial computed tomography (CT) of the left femur at an external facility revealed a left femoral fracture, prompting referral to our institution for definitive management of a suspected pathological fracture of the left femur. Since symptom onset, the patient has remained alert and oriented, with stable appetite, sleep, and bowel habits, and no notable weight fluctuations. Notably, the patient had a 17-year history of hepatosplenomegaly, with a confirmed diagnosis of GD established 4 years prior, at which time splenectomy was performed. Before admission, the patient had not undergone ERT or genetic testing. Chronic lower back pain was a long-standing complaint, with the current presentation precipitated by the left femoral fracture. The patient denied a history of epilepsy or limb motor dysfunction, with preserved visual and auditory acuity. No family history of hereditary disorders was reported.

## 3 Clinical findings

### 3.1 Imaging diagnosis

#### 3.1.1 Anteroposterior and lateral views of the left femur on DR

**Figure 1:**
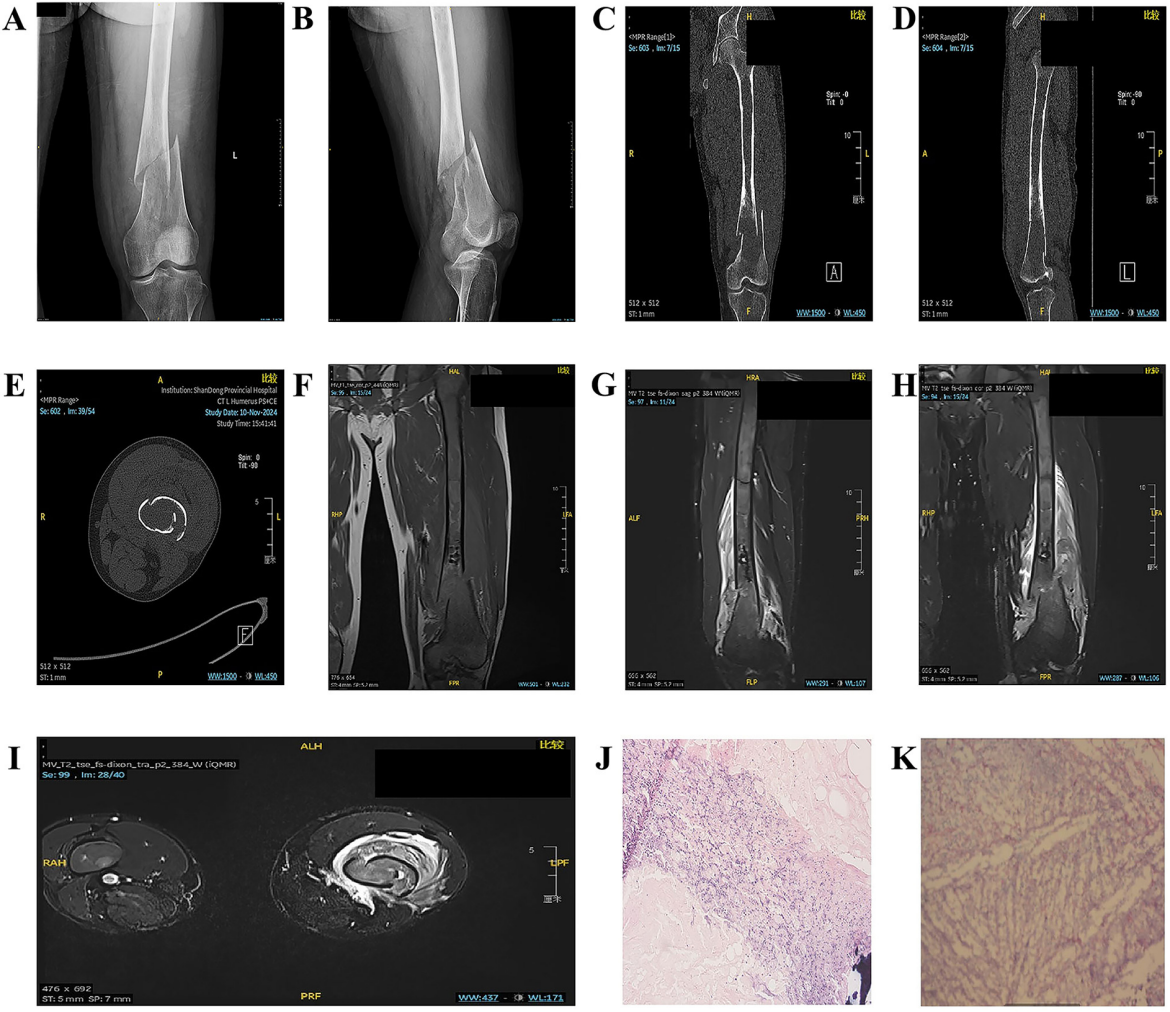
**(A,B)** Anteroposterior and lateral views of the left femur on DR. **(C–E)** Noncontrast 3D-CT scan of the left femur. **(F–I)** Noncontrast MR scan of the left thigh. **(J,K)** Observation results of paraffin-embedded sections of GD tissue.

Imaging of the left distal femur revealed heterogeneous osteopenia with cortical discontinuity and malalignment of the fracture fragments. The surrounding soft tissues demonstrated no abnormal radiodensity. These findings are consistent with a pathological fracture of the left distal femur (Figures 1A,B).

#### 3.1.2 Noncontrast 3D-CT scan of the left femur

Noncontrast computed tomography (CT) of the left distal femur revealed an oval osteolytic lesion with mild expansion and adjacent cortical thinning. The lesion lacked a well-defined sclerotic margin, and the overlying cortical bone was disrupted, resulting in malalignment of the fracture fragments. No intralesional calcifications were identified, and the surrounding soft tissues exhibited normal radiodensity. These findings are consistent with the imaging characteristics of a tumor-related pathological fracture. Further characterization with magnetic resonance imaging (MRI) is recommended to evaluate soft tissue involvement and define the extent of the lesion (Figures 1C–E).

#### 3.1.3 Noncontrast MR scan of the left thigh

Noncontrast magnetic resonance imaging (MRI) of the left distal femur revealed a cortical breach with displacement and malalignment of the fracture fragments. The femoral shaft medullary cavity, spanning from the intertrochanteric region to the distal metaphysis, exhibited heterogeneous patchy hypointensity on T1-weighted imaging and mild hyperintensity on fat-suppressed T2-weighted imaging (FS-T2WI), with poorly defined margins. A fusiform, irregular soft tissue mass with a mixed hyperintense signal on both T1WI and FS-T2WI encircled the mid-to-distal femoral shaft, with maximal prominence at the fracture site. Diffusion-weighted imaging (DWI) and apparent diffusion coefficient (ADC) maps revealed restricted diffusion within the mass. These findings, including the pathological fracture, are consistent with the imaging characteristics of a malignant bone neoplasm. Differential diagnoses include Ewing sarcoma and parosteal osteosarcoma, with histopathological confirmation pending (Figures 1F–I).

### 3.2 Pathological diagnosis

#### 3.2.1 Intraoperative frozen section pathological diagnosis

Histopathological examination of the left femoral lesion tissue revealed extensive degenerative changes and necrosis, accompanied by focal inflammatory cell infiltration. Foci of bone tissue were identified in scattered regions. Definitive diagnosis necessitates further evaluation via paraffin-embedded tissue sections.

#### 3.2.2 Pathological diagnosis of paraffin sections

Histopathological analysis of the left femoral lesion revealed extensive degenerative necrosis and focal inflammatory cell infiltration. Foci of bone tissue were identified in scattered regions, alongside sheets of histiocytes that were morphologically consistent with Gaucher cells. These findings align with the histopathological hallmarks of GD (Figures 1J,K).

### 3.3 Laboratory tests

Laboratory analysis revealed elevated serum alkaline phosphatase (ALP) levels, indicative of possible bone metabolic derangements or hepatosplenic involvement (5). Red blood cell (RBC) and hemoglobin (Hb) levels were below the reference range, indicative of anemia presumably secondary to bone marrow involvement. The prolonged prothrombin time (PT) and activated partial thromboplastin time (APTT) suggested coagulation dysfunction, potentially related to hepatosplenic abnormalities. Collectively, these findings reflect the multisystemic involvement inherent to GD. Notably, the patient exhibited thrombocytosis, a finding atypical for GD. Conventionally, GD patients develop secondary hypersplenism due to hepatosplenomegaly from glucocerebroside accumulation in the liver and spleen, which typically results in pancytopenia rather than thrombocytosis (6). Hypersplenism normally accelerates platelet destruction, resulting in thrombocytopenia. Given the patient's young age and splenectomy performed four years prior, physiological compensation would theoretically restore platelet counts to within normal ranges. Notably, however, the patient exhibited persistent thrombocytosis. This paradox may be attributed to dysregulated compensatory mechanisms or, potentially, the rare GBA mutations (A170H and A271V). Nevertheless, conclusive evidence remains insufficient, and longitudinal case data accumulation is warranted to inform future mechanistic investigations.

### 3.4 Five bone metabolism markers

**Figure 2:**
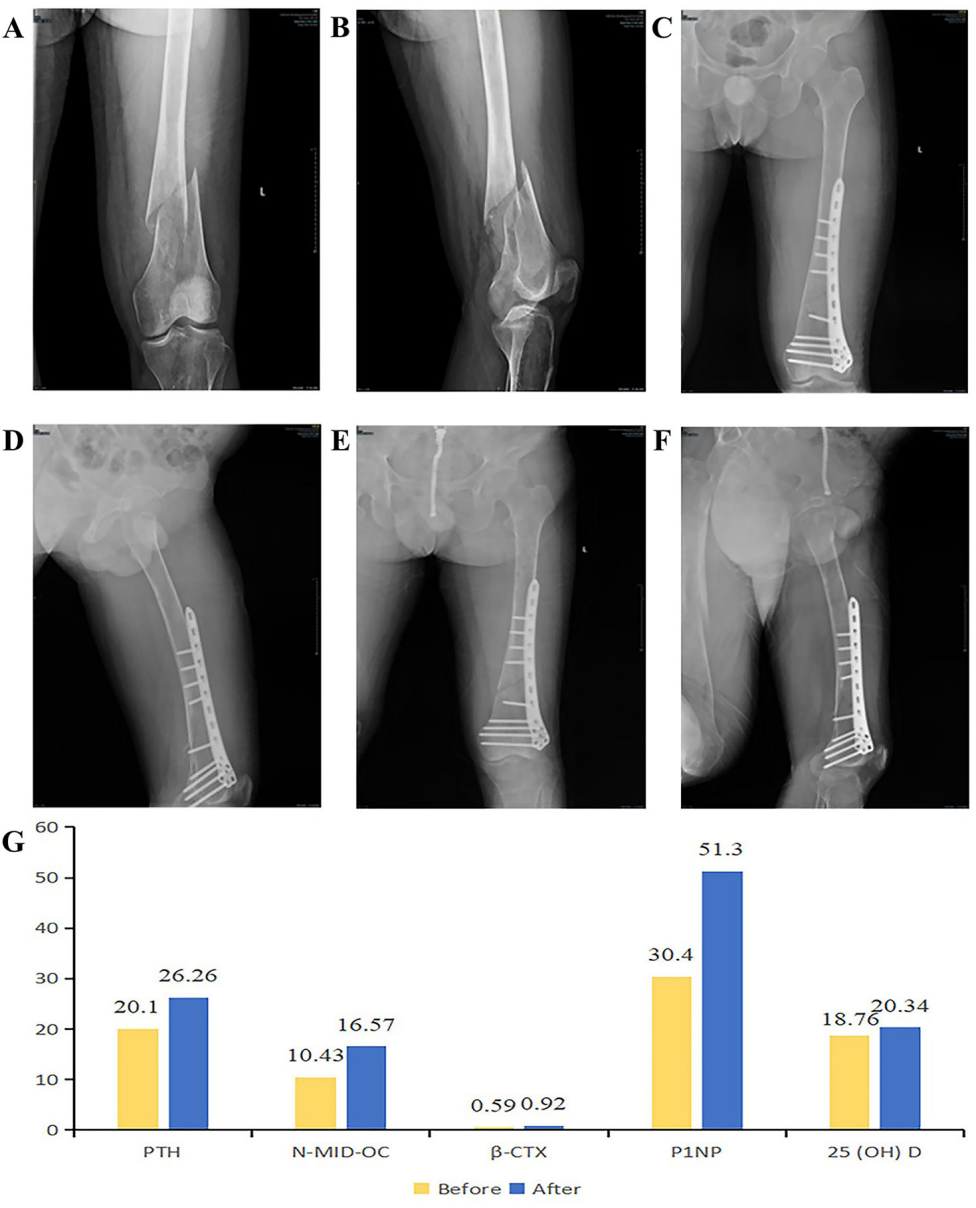
**(A,B)** fracture conditions before the operation. **(C,D)** After the operation, the fracture ends are in good alignment, and the fixation is firm. **(E,F)** Anteroposterior and lateral views of the left femur in the DR group during the follow-up examination. **(G)** Comparison of five bone metabolism indicators before and after surgery.

Laboratory analysis revealed that the N-terminal mid-fragment of osteocalcin (N-MID osteocalcin) was below the reference range, a finding indicative of diminished bone formation or dysregulated bone metabolism. Elevated beta-C-terminal telopeptide of type I collagen (β-CTX) levels suggest increased bone resorption, potentially linked to osteoporosis or other bone metabolic conditions. Notably, GD itself is a known contributor to skeletal pathology. Furthermore, 25-hydroxyvitamin D deficiency likely exacerbates bone metabolic derangements, thereby increasing the risk of fractures and skeletal deformities (7). These observations are potentially associated with the patient's chronic lower back pain and unexplained fractures (Figure 2G).

### 3.5 Glucocerebrosidase (GCase) activity assay

Determination of GCase activity remains the gold standard for diagnosing GD (8). The reference range for GCase activity is 1.19–22.23 μmol/L/h, whereas the patient's measured activity is 1.15 μmol/L/h, which is below the lower limit of normal. These results demonstrate significantly decreased GCase activity, which, when combined with clinical manifestations, confirms the diagnosis of GD.

### 3.6 Assay for the GD biomarker Lyso-GL-1

Lyso-GL-1 (lyso-glucocerebroside) serves as a specific biomarker for GD, with significantly elevated levels observed in affected patients. While the reference range for GBA activity is 1.19–22.23 μmol/L/h, the patient's measured activity of 1.15 μmol/L/h, although marginally below the lower limit, suggests a biologically meaningful reduction when contextualized with clinical findings. Measurement of Lyso-GL-1 levels is therefore critical for diagnostic support, particularly in patients with borderline GBA activity. The patient's Lyso-GL-1 concentration exceeded 400 ng/ml, further strengthening the reliability of the GD diagnosis.

### 3.7 Genetic testing

**Figure 3:**
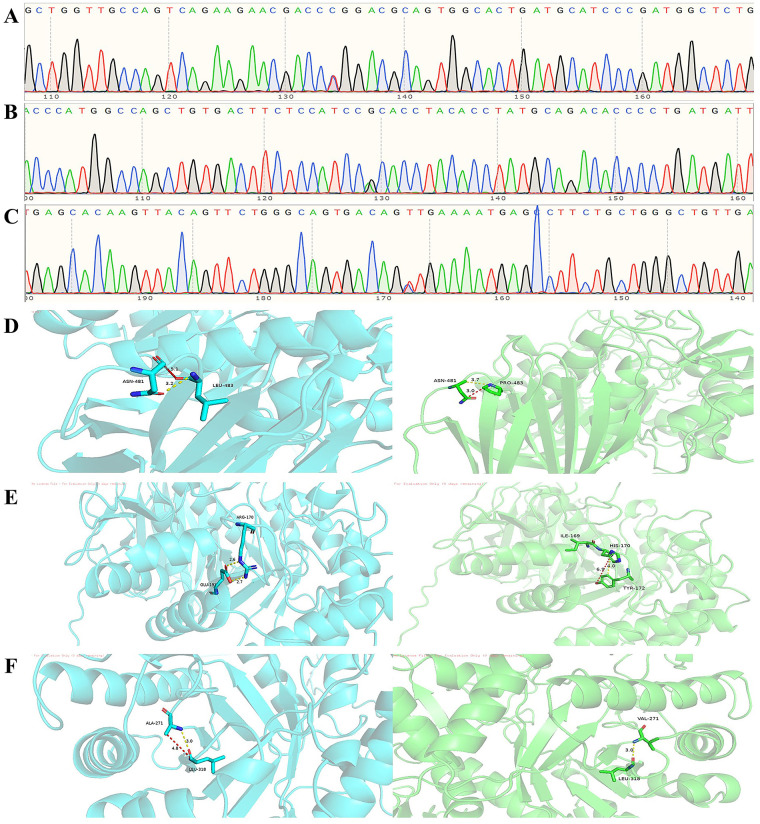
**(A)** Heterozygous exon 11 c.1448T>C (p.Leu483Pro) mutation. **(B)** Exon 6 c.509G>A (p.Arg170His) heterozygous mutation. **(C)** Exon 8 c.812C>T (p.Ala271Val) heterozygous mutation. **(D)** Molecular contacts of residue 483. **(E)** Molecular contacts of residue 170. **(F)** Molecular contacts of residue 271.

Genetic sequencing revealed that the patient was a carrier of the L444P pathogenic mutation and the A170H variant of uncertain significance (VUS). To date, two cases in the literature have reported the A170H mutation in GD patients. Structural prediction software (MutationTaster) suggested that this variant may impair protein function. Additionally, the patient harbors the A271V mutation, which lacks established clinical significance and is absent from GD-specific genetic databases. Nevertheless, its potential association with GD cannot be ruled out (Table 1; Figures 3A–C).

**Table 1:** Results of genetic testing.

Gene and transcript information	Related diseases and inheritance patterns	Chromosomal location and genetic mapping	Variant sites and mutation analysis	Exon/Intron	Heterozygosity	Highest Allele frequency in gnomAD	Variant classification	Origin of the variant
GBA NM_001005741.3	GD (AR)	chr1:155205043	c.1448T>C p.Leu483Pro	exon11	Heterozygous	0.0034	Pathogenic	–
GBA NM_001005741.3	GD (AR)	chr1:155208387	c.509G>A p.Arg170His	exon6	Heterozygous	2.6e-05	Likely pathogenic	–
GBA NM_001005741.3	GD (AR)	chr1:155207319	c.812C>T p.Ala271Val	exon8	Heterozygous	–	Variant of uncertain significance	–

## 4 Therapeutic intervention

### 4.1 Resection and curettage of left femoral lesions with open reduction and internal fixation (ORIF)

ORIF is relatively simple to perform. Directly fixing the fracture fragments with screws, it can achieve rigid internal fixation, reduce micromotion at the fracture end, and thus be more conducive to fracture healing. In addition, the postoperative infection rate of ORIF is approximately 5%–10%, which is relatively low. Although intramedullary nailing may be more advantageous in cases of poor bone quality, the patient's fracture is located in the distal femur, where the medullary cavity is wide and adjacent to the joint. Moreover, GD itself can cause bone abnormalities, so intramedullary nailing may have difficulty providing sufficient stability. Moreover, reaming is required during intramedullary nailing, which may increase the risk of fat embolism. On the basis of the above factors, we ultimately chose ORIF for treatment.

A 20-cm longitudinal lateral incision was made along the middistal left femur. The medullary canal was occupied by reddish granulation tissue. A representative lesion sample was submitted for intraoperative frozen section analysis, which revealed extensive degenerative necrosis, focal inflammatory cell infiltration, and focal bone tissue within the left femoral lesion. Further evaluation via paraffin-embedded tissue sections is pending. The surgical field was irrigated with sterile distilled water, and the femoral fracture fragments were reduced. A 9-screw lateral femoral plate (Hebei Ruihe, China) was applied for internal fixation. Preoperative and postoperative radiograph comparisons are depicted in the figure (Figures 2A–D).

### 4.2 Enzyme replacement therapy (ERT)

Imiglucerase is the primary agent utilized for ERT in GD. As a recombinant form of GCase generated via genetic recombination technology, it shares structural and functional homology with native human GCase (9). This enzyme selectively binds to and hydrolyzes accumulated glucocerebroside in GD patients, thereby mitigating its intracellular accumulation.

Following surgery, the patient commenced a recommended ERT regimen with Imiglucerase (400 U/vial). A cyclic dosing protocol was instituted, with each complete cycle lasting two months. During the first month of each cycle, 7 vials were administered, followed by an adjustment to 8 vials in the subsequent month. This dosing pattern was repeated cyclically, with the total monthly dose delivered via a single injection.

## 5 Follow-up and outcomes

### 5.1 Imaging diagnosis

Three months after surgery, the patient's fracture line appeared blurred (Figures 2E,F). Six months postoperatively, although the patient's fracture had not yet met the criteria for clinical union, the patient was able to perform movements such as hip flexion, hip extension, knee flexion, and knee extension. The patient could stand independently and walk with bilateral crutches, with a basic recovery of daily living and work abilities.

### 5.2 Five bone metabolism markers

Follow-up analyses revealed significant improvements in five bone metabolism markers at 3 months after ERT. N-MID osteocalcin increased from 10.43 ng/ml to 16.57 ng/ml, reflecting enhanced osteoblastic activity and favorable fracture healing progression. β-CTX levels rose from 0.59 ng/ml to 0.92 ng/ml, potentially indicating the patient's status in the early phase of fracture healing. During this stage, increased bone resorption represents a normal physiological process, as osteoclasts are necessary to clear damaged bone tissue and facilitate neogenesis. However, persistent elevation of β-CTX may signify excessive bone resorption or deterioration of bone metabolic derangements. In the context of a patient's GD, continuous surveillance of these biomarkers remains critical. Notably, the total procollagen type I N-terminal propeptide (P1NP) level increased significantly from 30.40 ng/ml to 51.30 ng/ml, reflecting active bone repair and remodeling processes and confirming the efficacy of ERT. Furthermore, the 25-hydroxyvitamin D level increased from 18.76 ng/ml to 20.34 ng/ml, indicating improved overall bone health. Collectively, these findings demonstrate favorable postoperative bone healing in this patient, with ERT achieving substantial therapeutic outcomes (Figure 2G). Six months later, all indicators tended to be stable. Among them, the level of β-CTX decreased from 0.92 ng/ml to 0.691 ng/ml, indicating that osteoclast activity was inhibited, the bone resorption process was weakened, the osteogenic effect was enhanced, and fracture healing was in the progressive stage.

## 6 Discussion

The GBA gene resides on human chromosome 1q21 and consists of 11 exons. The mutational profiles and prevalence of this gene exhibit pronounced racial and ethnic disparities. The L444P mutation (p.Leu444Pro) represents one of the most frequently observed variants, with a significantly higher allelic frequency documented in Asian populations (10). In Chinese patients, the L444P mutation accounts for approximately 40% of cases. The L444P mutation in this patient was the L444P mutation. The N370S mutation is the most prevalent mutation type in Type I GD (11) and is also found at the highest frequency among Ashkenazi Jewish populations. In the genetic testing report of this case, we also identified the A170H missense mutation. However, its potential pathogenicity has only been suggested in a few reports (12, 13), and the evidence remains inconclusive. Notably, the A170H variant is predominantly associated with hereditary methemoglobinemia. In future clinical practice or research, additional GD cases harboring the A170H variant should be identified, and the comprehensive clinical data, diagnostic and therapeutic workflows, and laboratory findings from these cases could serve as critical references for investigating the pathogenic potential and clinical relevance of the variant in GD. This would facilitate a more comprehensive understanding of the A170H variant within the context of GD.

Upon mutation of Leu-483 to Pro-483, the hydrophobic isobutyl side chain of the wild-type Leu residue is replaced by the cyclic proline side chain. The interatomic distances between Pro-483 and adjacent regions decrease from 5.1 Å and 3.2 Å to 3.7 Å and 3.0 Å. Theoretically, this distance reduction could directly enhance van der Waals forces. However, the rigid cyclic structure of proline imposes constraints on the backbone conformation, leading to increased local steric hindrance and diminished flexibility of hydrophobic interactions, which in turn reduces the spatial adaptability of van der Waals forces. Consequently, the net effect of the L444P mutation on van der Waals forces remains ambiguous and may necessitate further structural dynamics analysis for clarification. Notably, the conformational rigidity of proline (Pro) may hinder GCase folding or active site conformational changes, potentially serving as a primary mechanism underlying GD pathogenesis in the L444P mutation. Concerning the A170H mutation, the positively charged guanidino group of the wild-type arginine (Arg) residue is replaced by the imidazole group of histidine (His). The significant reduction in side-chain volume leads to increased local interatomic distances; as illustrated in the figure, these distances expand from 2.6 Å and 2.7 Å to 6.9 Å and 4.0 Å, thereby weakening van der Waals forces. Notably, the imidazole group may form hydrogen bonds or π-π stacking interactions at physiological pH, partially offsetting the loss of hydrophobic interactions. However, decreased side-chain rigidity of the mutant residue may disrupt the hydrophobic microenvironment of isoleucine (Ile)-169 in the adjacent polypeptide backbone, compromising local structural stability and potentially impairing GCase function. For the A271V mutation, the methyl side chain of alanine (Ala) is substituted by the isopropyl group of valine (Val). Notwithstanding the increase in side-chain volume, the interatomic distance between Val-271 and adjacent regions remains unchanged at 3.0 Å postmutation. This observation suggests that the bulkier isopropyl group may facilitate more compact local packing through side-chain conformational adjustments or adaptive displacement of neighboring atoms, thereby preserving the stability of critical interatomic distances. The conservation of leucine (Leu)-318 further implies the functional importance of this residue in maintaining global structural integrity. Notably, as no cases linking the A271V mutation to GD have been documented to date, its clinical significance remains undefined. If future studies identify GD cases associated with the A271V mutation, the findings from this research could provide valuable insights (Figures 3D–F).

The distinct skeletal manifestations in GD patients significantly increase both surgical and postoperative complication risks, thereby limiting the use of internal fixation surgery for fractures in this population. Skeletal involvement in GD typically encompasses osteoporosis, osteonecrosis, bone infarction, and pathological fractures, which arise from bone marrow infiltration by Gaucher cells, compromised osseous blood supply, and metabolic derangements (14). The bone healing capacity of patients with GD is generally impaired following surgery. Furthermore, GD patients frequently exhibit hematologic abnormalities, including hypersplenism, thrombocytopenia, and anemia, which increase the risk of intraoperative hemorrhage and postoperative infections. Suboptimal bone quality in this population may also contribute to internal fixation failure or recurrent fractures. In the present case, the patient faced a high risk of postoperative osteonecrosis due to the inherent pathological features of GD. However, given the patient's planned continuous ERT—a modality established to increase bone metabolism—their bone healing capacity is anticipated to surpass that of GD patients not receiving ERT. Given these considerations, we elected to perform curettage of the left femoral lesion, fracture reduction, and internal fixation.

Additionally, we herein discuss the classification of GD in this patient. The L444P mutation is the most common allelic mutation among Chinese patients with GD and is closely associated with neurological manifestations. The L444P variant is commonly linked to type III GD, and numerous cases initially diagnosed with type I GD have been reclassified as type III due to the delayed onset of neurological symptoms, underscoring the variant's association with progressive neurodegeneration. Given the patient's young age, the nervous system may remain unaffected, or physiological compensatory mechanisms may have mitigated the onset of overt neurological symptoms, potentially coupled with subclinical disease progression. Long-term follow-up is imperative to closely monitor and evaluate neurological manifestations, track disease progression, and promptly adjust the treatment plan as needed.

Finally, we advocate for innovation in the diagnostic and therapeutic algorithms for GD. Dry blood spot (DBS) triple testing—encompassing biomarker analysis, enzymatic activity assays, and genetic sequencing—has emerged as a valuable tool for high-risk GD screening. In this case, for example, while GCase activity showed no significant reduction, the doubling of biomarker values substantially strengthened the diagnostic confidence for GD. Minimizing misdiagnosis and missed diagnosis rates represents a critical milestone in the early management of GD and other rare diseases.

Moving forward, we anticipate the reporting of additional GD cases harboring novel gene mutations, as such contributions will furnish a robust theoretical basis for the advancement of innovative treatment protocols in GD.

## Data Availability

The original contributions presented in the study are included in the article/Supplementary Material, further inquiries can be directed to the corresponding authors.
